# An audit experiment to investigate the “war on cops”: a research note

**DOI:** 10.1007/s11292-021-09458-x

**Published:** 2021-03-18

**Authors:** David S. Kirk, Marti Rovira

**Affiliations:** Department of Sociology & Nuffield College, University of Oxford, 1 New Road, Oxford, OX1 1NF UK

**Keywords:** Audit study, Discrimination, Employment, Experiment, Hiring, Police, Police brutality, Police violence, Police use of force, Stigma

## Abstract

**Objectives:**

This study examines whether former police officers are stigmatized in the labor market, particularly following social unrest from lethal police violence.

**Methods:**

We conduct an experimental audit study, both before and after heightened unrest from police violence. For service-related job openings, we compare the likelihood of getting an affirmative response from a prospective employer to a job application from a fictitious former police officer (the treatment condition) to the response to one of two control conditions: a former firefighter or a former code enforcement officer.

**Results:**

We do not find evidence that former police officers are discriminated against in the labor market. This finding holds in periods characterized by relatively little social unrest due to police violence as well as periods of heightened protest activity.

**Conclusions:**

At least with respect to the labor market for certain service-related professions, former police officers do not appear tainted by any stigma associated with their prior profession.

**Supplementary Information:**

The online version contains supplementary material available at 10.1007/s11292-021-09458-x.

## Introduction

Among the most controversial arguments considered in recent years by criminologists has been Heather Mac Donald’s (2016) assertion that the public reaction to the shooting death of Michael Brown by Ferguson (MO) police officer Darren Wilson in August 2014 triggered a “war on cops.” This so-called war has purportedly led to the physical and verbal assault of numerous police officers, and led demoralized officers to essentially abandon their duties and let crime proliferate for fear of being videotaped engaging in aggressive, proactive policing (Mac Donald 2016). More recently, Mac Donald (2020) vehemently argued that this “Ferguson Effect” has repeated itself following the social unrest spurred by the killing of George Floyd by Minneapolis police officer Derek Chauvin in May 2020.

If there truly is a “growing crusade against law enforcement” (Mac Donald 2016, p. 4), current and former police officers could be tainted in their social relations beyond their professional duties, even in the labor market when they seek second jobs or another career. Policing is a profession with a high rate of turnover, with nearly 8% of police officers voluntarily resigning from the job each year, and another 1% retiring (Wareham, Smith, and Lambert 2015). Departures from the profession were even more acute in the wake of social unrest following the death of George Floyd. Particularly for those officers who resign, but even for those who retire, it is often necessary to find another career. Yet do former officers stand a chance at gainful employment outside of law enforcement given the purported “war on cops?”

In this research note, we seek to demonstrate the possibilities of experimental audit methods for investigating public reactions toward police officers. Public opinion data reveals heightened negative sentiment toward the police following police violence as well as increasing acknowledgement of, and anger over, inequitable treatment of Black individuals by the police (Pew Research Center 2020). Accordingly, we are interested in measuring the tangible consequences of these adverse public reactions to the lives of police officers. Specifically, we examine whether officers who have left the police force are stigmatized in the labor market, effectively reducing their life chances. We implemented an audit test in two periods, before and after the killing of George Floyd. With the important caveat that, because of the COVID-19 pandemic, the labor market in the year leading up to Mr. Floyd’s death was dissimilar to the labor market after his death, we seek to determine if former police officers are any more or less stigmatized in the labor market after high-profile incidents of police violence.

## Research design

Audit studies are a type of field experiment used to test for discriminatory behavior. In such experiments, one characteristic of individuals distinguishes the treatment and control groups, and the experiment is designed to gauge whether the groups are treated differently because of this characteristic. For instance, in one of the most common applications in criminology, two otherwise similar fictitious job applicants apply for the same jobs, with the applicants distinguished by the presence of a criminal record (see, e.g., Pager 2003). Researchers then measure differential responses to the fictitious job applications—e.g., an invitation to interview for the job—as an indicator of discrimination based on the mark of a criminal record. Through the creation of equivalent applicant backgrounds across the fictitious job applicants, audit methods reduce the problems of omitted variables and selection bias often found in studies of discrimination.

Whereas much of the application of audit methods in criminology focus on the stigma of a criminal record, here, we turn the lens to the potential stigma of being a police officer. With widespread social unrest from police violence, police as a whole may become stigmatized. Affiliation with the police may, to some individuals, signal a blemish of individual character (Goffman 1963), for instance that the individual is racist, overly aggressive, and unable to treat people with fairness and respect. Such qualities may be viewed as undesirable in the labor market. Accordingly, using a matched pairs design, we compare the likelihood of getting an affirmative response from a prospective employer to a job application from a fictitious former police officer (the treatment condition) to the response to one of two control conditions: a former firefighter or a former code enforcement officer. We signal the nature of prior employment through the job experience listed on the applicant’s resume and job application. We chose firefighting and code enforcement as control conditions given their similarity in requisite professional skillsets, a choice we return to in our concluding section.

We conducted our audit test at two separate time points: once in a period absent social unrest from police violence, and second during the heightened unrest following the killing of George Floyd on May 25, 2020. Under normal circumstances, this before-after design might allow for a natural experiment to examine whether incidents of publicized police brutality produced a change in the amount of differential response to former police officers in the labor market. However, the second time period of our data collection coincided with the fallout of the COVID-19 pandemic and the corresponding damage to the labor market, so the results should be interpreted cautiously.

We pre-registered the design of our study on Open Science Framework (OSF), where our data and code is archived (https://osf.io/p8je9/).

### Sampled cities

We sampled employers in two Northeastern cities: Boston and Philadelphia. Resource constraints and the COVID-19 lockdown prevented us from extending the study to the Midwest, South, and West. To select these two cities, our sampling frame included the primary city in each metropolitan area in the Northeast that had a population size greater than 1 million. We only sampled cities in large metropolitan areas in order to ensure a sufficiently large number of potential jobs for which to apply, and to avoid diluting the job market with fictitious applications. Using information from the Fatal Encounters data repository (http://www.fatalencounters.org/), we computed the rate of deaths by the police in each large city. We then ranked and split the cities into two groups at the median: a “low” police violence group and a “high” police violence group. We then randomly selected one city from the “low” group (Boston) and one from the “high” group (Philadelphia).

### Characteristics of fictitious applicants

In developing the fictitious resumes for the experiment, we created 12 distinct profiles that varied by profession (police, firefighter, or code enforcement), gender (male or female), and race (Black or White). We signaled race and gender through first and last names.^1^ When applying for a given job, race and gender of the two applicants were identical, and we randomly determined which applicant race, gender, and control condition to use. For all job openings, one of the applicants had most recently been employed as a police officer, with the other applicant most recently a firefighter or a code enforcement officer.^2^

We meticulously developed resumes in order to ensure similarity across background characteristics, but without exactly matching resume information and style. For residential addresses, we used real street names but fictitious street numbers in two different neighborhoods in the given city with similar race-ethnic compositions and poverty levels. For educational background, we selected public high schools characterized by similar race-ethnic composition and the percent of students qualifying for reduced-priced or free school meals. All applicants graduated from high school in May or June 2013. With an approximate birth year of 1995, necessarily, our experiment is focused on individuals who had relatively brief stints as police officers, roughly 3 years, before separating from the profession.

Prior to becoming a police officer, firefighter, or code enforcement officer, our fictitious applicants worked as delivery drivers and as sales clerks/cashiers in retail businesses. Specific elements of the resumes (e.g., addresses, high school attended, graduation date, prior jobs, and skills) were randomized for each job opening.

### Characteristics of targeted jobs and employers

Our design is a version of an audit study known as an online correspondence test, in which we applied to online job advertisements. We submitted fictitious applications to job openings advertised in the Boston and Philadelphia metropolitan areas on Indeed.com and Craigslist.org. We applied to four different types of jobs: (1) skilled trades, such as electricians or painters; (2) drivers; 3) retail sales; and (4) office and customer support. We excluded jobs related to security and law enforcement. We expected that for these four occupations, former police, firefighters, and code enforcement officers would be similarly qualified. Hence, if there is preferential treatment of one group over another, it may be due to the stigma of their prior profession rather than their actual qualifications.

We targeted newly listed openings occurring within the preceding week. We did not apply to jobs requiring a social security number. We only applied to one position per employer. For companies with multiple branches and sites, we only applied to one position per branch. Based on these criteria, we selected jobs by convenience, as opposed to randomly sampling from a listing of all job openings matching our eligibility criteria.

When applying for jobs, we randomized the order of the application—i.e., whether the first application was sent from the treatment condition (i.e., police) or control (firefighter or code enforcement). We submitted the second application approximately 3 hours after the first one. For openings found on Craigslist, we submitted applications via email, including a brief cover email and an attached resume. We designed the content of the emails to be similar but not exact across the treatment and control cases, and randomized which version was sent by the treatment vs. control condition. For Indeed.com openings, we submitted job applications either through its platform or through a linked company site. These submissions typically required completion of an online form that duplicated the content of our attached resume. If a cover letter was required, we replicated the contents of the emails.

### Outcome variable

Each of the 12 different applicant profiles used in the study had an email account and a unique phone number, which could receive texts and voicemails. Our outcome variable measures whether the fictitious applicant received an affirmative response to the application to set-up an interview or more informal requests to discuss the job. We excluded immediate auto-generated responses acknowledging receipt of the application. If an applicant received an affirmative response, we set the value of the outcome variable equal to one. For non-responses or negative responses, we set our outcome variable equal to zero.

### Implementation

As noted, we implemented our experiment at two time points, prior to and following the killing of George Floyd on May 25, 2020. Data from the Crowd Counting Consortium (https://sites.google.com/view/crowdcountingconsortium/home), a collaborative crowd-sourcing effort led by political scientists Erica Chenoweth and Jeremy Pressman, reveals that more than 5000 different anti-racism and anti-police-brutality protest events took place nationwide in the first 6 weeks after Mr. Floyd’s death, with between 15 and 26 million individuals participating in these protests, including large numbers in Philadelphia and Boston (Buchanan, Bui, and Patel 2020; Putnam, Pressman, and Chenoweth 2020). Scholars and experts have characterized the combination of the size, intensity, and frequency of the protests as “unprecedented” (Putnam, Chenoweth, and Pressman 2020), underscoring the potential for differential treatment of the police across the two time periods of our study.

Our first implementation period took place between late May 2019 and March 2020, and was discontinued because of the COVID-19 pandemic. Termination of the data collection occurred on March 11, 2020, roughly 1 week prior to the first stay-at-home order in the USA, in the San Francisco area, and about 2 weeks prior to widespread issuance of stay-at-home orders. Our sample size of jobs in this period equals 605, with 1,210 applications.

We re-launched our data collection on June 4, 2020, 10 days after the killing of George Floyd. The data collection ended on July 16, 2020. In May of that year, both Massachusetts and Pennsylvania began reopening their respective economies after the initial lockdown from COVID-19. However, the necessity of social distancing and minimizing the spread of the virus meant that the state of the economies across our two time periods differed substantially. Our sample size of jobs from this period equals 212, with 424 applications.

### Analysis plan

In the analysis to follow, we first present descriptive results of the proportion of applications for which police, fire, and code enforcement applicants received an affirmative response, separately for our two time periods. We then use McNemar’s (1947) test to examine statistical inferences. In an online Technical Appendix, we supplement these analyses by estimating linear probability models of the likelihood of an affirmative employer response, to facilitate examination of possible heterogeneous effects by applicant race and gender.

McNemar’s test, which is applied to a 2 × 2 contingency table, is a Chi-square test of goodness of fit for paired nominal data that can be used to compare the distribution of employer responses expected under the null hypothesis to the actual observed responses. Table 1 illustrates the test. In this table, *n*_*ab*_ represents the count of the number of cases in a given cell. The proportion corresponding to the count then equals to the following: *p*_*ab*_ = *n*_*ab*_/*n*. The first subscript represents the outcome for the control condition in the rows (i.e., prior employment as a firefighter or code enforcement officer). The second subscript denotes the outcome for the treatment condition (i.e., prior employment as a police officer). An affirmative response equals 1 and a negative response equals 0.

**Table 1:**
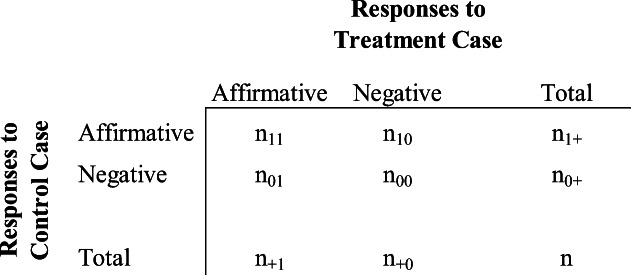
2 × 2 contingency table for McNemar’s test

Our focus with McNemar’s test is on the discordant pairs, *n*_*10*_ and *n*_*01*_. More specifically, McNemar’s test assesses the null hypothesis of marginal homogeneity: π_1+_ = π_+1_, which is equivalent to testing whether the difference between the two discordant proportions equals 0—i.e., π_10_ = π_01_ (Vuolo, Uggen, and Lageson 2016). Accordingly, we are assessing whether the proportion of job applications for which a former police officer received an affirmative response from the employer but not the former firefighter is the same as the proportion of job applications for which a former firefighter received an affirmative response from the employer but not the former police officer. The test statistic is:

$$ {\chi}^2=\frac{{\left({n}_{10}-{n}_{01}\right)}^2}{\left({n}_{10}+{n}_{01}\right)} $$

A statistically significant Chi-square test would provide evidence to reject the null hypothesis, interpreted to mean that the likelihood of an affirmative response to a job application differs between the treatment (i.e., former police officer) and the control condition (former firefighter or code enforcement officer).

## Results

**Fig. 1:**
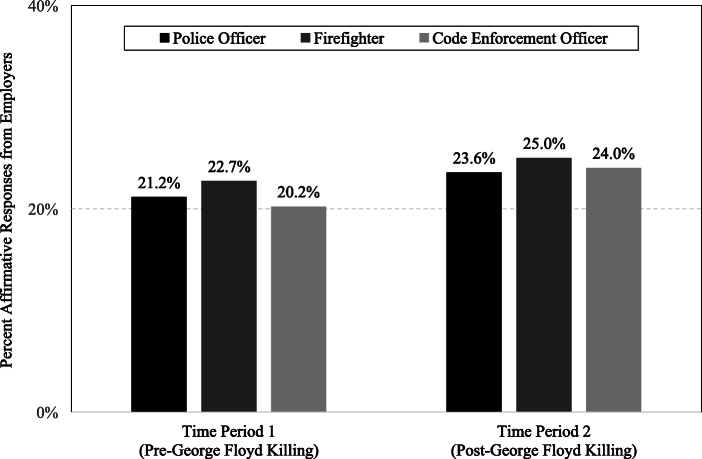
Percent of affirmative employer responses to job applications, by former profession and time period

Figure 1 displays employer response rates to job applications from the three experimental conditions across the two time periods. Visually, these results reveal fairly consistent response rates across experimental conditions, ranging from approximately 20 to 23% in the first time period and 23 to 25% in the second. The police do receive the lowest response rate among the three groups in the second time period; however, it is not substantially lower than the others. That said, these descriptive results do not factor in the paired nature of the data.

In Table 2, we present results of McNemar’s tests of our paired data from job applications submitted prior to the COVID-19 lockdown and the killing of George Floyd (May 2019 to March 2020). The top panel (2A) shows the comparison between response rates when the applicant is a former police officer vs. a former firefighter (*N* = 308). For the vast majority of openings (224/308 = 72.7%), both the police officer and the firefighter either received a negative response to their job application or a non-response. In 15.6% of the openings (48/308), both experimental conditions received an affirmative response from the employer.

**Table 2:**
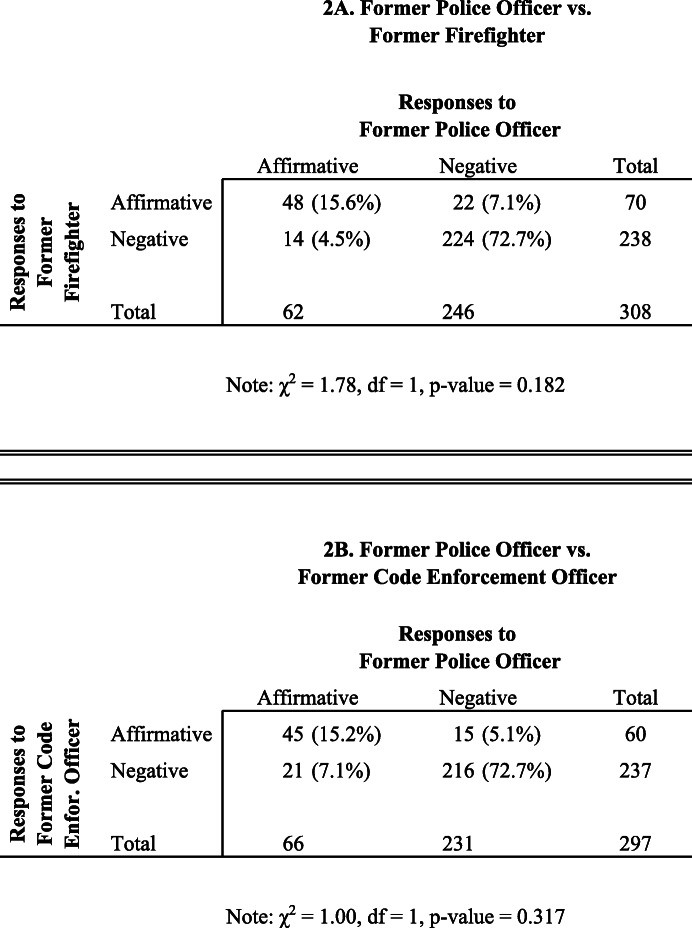
McNemar’s test of the distribution of employer responses by experimental condition, time period 1 (pre-George Floyd killing). A. Former police officer vs. former firefighter. B. Former police officer vs. former code enforcement officer

As noted, for McNemar’s test, focus is on the discordant cells. For 7.1% of the job openings (22/308), the firefighter received an affirmative response but the police officer received a negative one. For 4.5% of the openings (14/308), the police officer received an affirmative response whereas the firefighter did not. The Chi-square test (1.78, df = 1, *p* value = 0.182) indicates that we lack evidence to reject our null hypothesis. Hence, we do not find evidence that former police are treated differently relative to former firefighters.

The bottom panel (2B) in Table 2 presents similar results for the comparison between former police and code enforcement officers (*N* = 297). For 5.1% of the job openings (15/297), the code enforcement officer received an affirmative response, but the police officer received a negative one. For 7.1% of the openings (21/297), the police officer received an affirmative response whereas the code enforcement officer did not. The difference is not statistically significant.

Table 3 displays results of McNemar’s test for the job applications submitted during the period immediately following the killing of George Floyd in May 2020. As stated, given the widespread reaction to the killing of Mr. Floyd, in the second version of the experiment, we sought to examine employer responses to former police officers in the context of widely publicized police violence and subsequent social unrest.

**Table 3:**
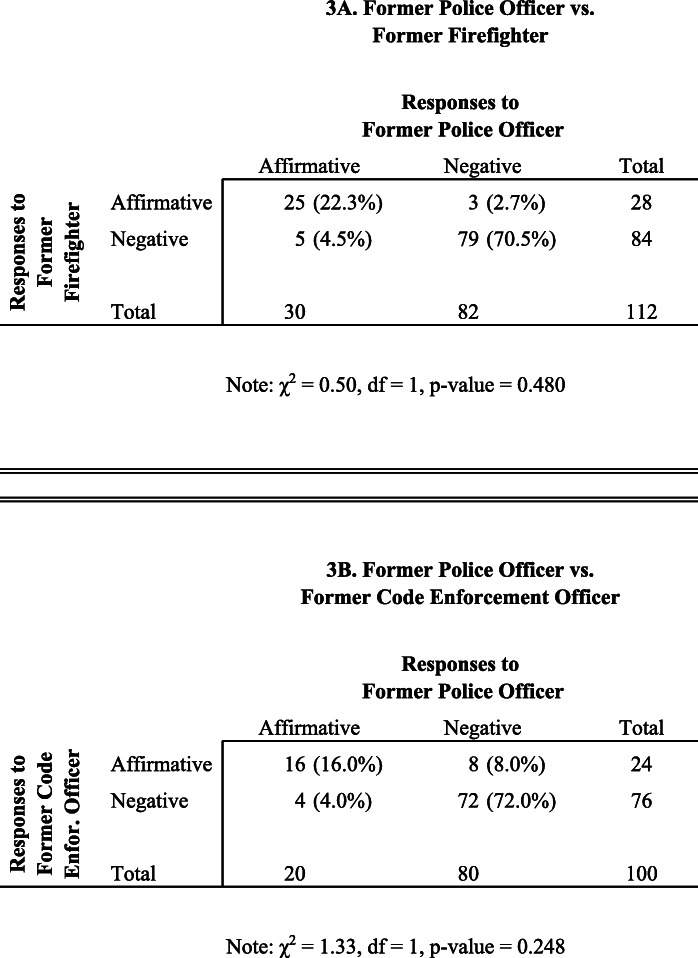
McNemar’s test of the distribution of employer responses by experimental condition, time period 2 (post-George Floyd killing). A. Former police officer vs. former firefighter. B. Former police officer vs. former code enforcement officer

In the top panel of the table (3A), we see that for three of the 112 job openings (2.7%), the former firefighter received an affirmative response, but the police officer received a negative one. Similarly, for five of the 112 openings (4.5%), the former police officer received an affirmative response, but the firefighter did not. The Chi-square value nearly equals zero (0.50, df = 1, *p* value = 0.480), and we find little evidence that former police officers are treated differently than former firefighters in the labor market, even in a period marked by widespread social unrest against police brutality.

In the bottom panel (3B), we find more variation in discordant responses, but still not enough to yield conclusive evidence about different treatment of former police officers relative to code enforcement officers. Our Chi-square test equals 1.33 (df = 1), with a *p* value of 0.248.

In the Technical Appendix, we display results of linear probability models (LPMs), which, as expected, are highly consistent with the results of McNemar’s tests (see Table A.1, Model 1). With the LPMs, we also examine heterogeneity in our findings by race and gender by including interaction terms between our treatment condition and these applicant characteristics. With one exception, an interaction between race and time period, none of the interactions are statistically significant. This general lack of heterogeneity may result from the fact that our analysis, particularly in the second time period, is underpowered. Hence, further examination of possible heterogeneity by race and gender is warranted in future research.

## Discussion

In this study, we augmented audit methods in order to conduct a novel test of the potential stigma of being a (former) police officer. Audit tests capture the “real world” behavior of employers while at the same time help reduce the problems of omitted variables and selection bias endemic to many studies of discrimination. Audit methods can provide unbiased estimates of discrimination to the extent that treatment and control groups are otherwise equivalent except for the one characteristic under investigation, in this case prior profession.

We chose firefighting and code enforcement as control conditions given their similarity in skillsets to police officers, at least with respect to the applicability of skills for our four sampled job categories. They are front-line public sector professions requiring responsiveness to public concerns and requests for assistance. Police officers, of course, develop different skills than firefighters and code enforcement officers through training and on-the-job performance, but we specifically sampled professions that do not necessitate those highly specialized skills, such as knowledge of criminal code or firearms training. Accordingly, we sought to make our fictitious applicants similarly qualified for the jobs for which they applied, so that the reason for a difference in the callback rate was not due to qualifications. That said, the extent to which we detect any discrimination against the police depends upon our choice of control conditions, and we might detect different levels of discrimination if we compare police against otherwise equivalent individuals with some other kind of professional background than firefighting and code enforcement.

A possible limitation of our study is the convenience sampling strategy we employed. We applied to the most recently posted job openings matching our defined eligibility criteria. The implication is that our results may not fully generalize to the population of jobs in a labor market.

Acknowledging caveats and potential limitations, in this study, we do not find evidence that former police officers are hindered in the labor market for service-related jobs in sales, customer service, transportation, and skilled trades. This finding holds even in the context of the aftermath of George Floyd’s killing, although we suggest that comparisons between the time periods before and after Mr. Floyd’s death be interpreted cautiously because the post-period coincided with disruption to the labor market from the COVID-19 pandemic.

How should we interpret our null findings? A non-significant difference in the callback rate between the former police officer and either of the control conditions could reflect the fact that ex-police are not, in fact, stigmatized in the labor market by the nature of their prior profession (i.e., evidence for our null hypothesis). Alternatively, we may be unable to detect discrimination against the police in our data, perhaps because our analysis, particularly in the period following George Floyd’s death, is underpowered, or our data is otherwise insensitive to detecting true stigmatization (see Dienes 2014; Benjamin et al. 2018). Further research is necessary in order to adjudicate between these possible interpretations of our null finding. Additionally, whereas our Technical Appendix displays some initial analysis of heterogeneous effects by applicant race and gender, we recommend further research to more thoroughly examine whether the null finding is consistent across different applicant attributes and also location.

In summary, social scientists have characterized the scale of the anti-racism and anti-police-brutality protests following George Floyd’s death as unprecedented (Putnam, Chenoweth, and Pressman 2020). However, these protests did not appear to lead to any negative implications for former police in the labor market. Hence, whereas Mac Donald (2016) has asserted that there is a “growing crusade” against the police, at least with respect to the labor market for former officers, we do not yet find evidence of any such crusade.

## Supplementary information

ESM 1 (DOCX 115 kb)

## Footnotes

1 (1) Black male: Tremayne Jefferson, Darnell Mosley, and Jabari Washington; (2) Black female: Janae Booker, Erykah Jackson, and Shanice Jefferson; (3) White male: Ethan Becker, Jake Meyer, and Ryan Walsh; (4) White female: Emily Decker, Katelyn Hartman, and Claire McGrath.

2 Code enforcement officers are public sector employees whose primary duties are ensuring compliance with local government codes and ordinances.
