# Supplementary material for: An audit experiment to investigate the “war on cops”: a research note
Source: J Exp Criminol. 2021 Mar 18;18(3):569–80. doi: 10.1007/s11292-021-09458-x (PMC7971355; doi:10.1007/s11292-021-09458-x)
Supplement: Supplementary file 1 — (DOCX 115 kb) [file 11292_2021_9458_MOESM1_ESM.docx]

**An Audit Experiment to Investigate the “War on Cops”:**

**A Research Note**

**David S. Kirk & Marti Rovira**

**Technical Appendix**

In this appendix, we estimate the difference in the response rate between our treatment condition (i.e., former police) relative to our control conditions (i.e., former firefighter or code enforcement officer) using linear probability models (LPMs). One reason to estimate LPMs was to facilitate examination of possible heterogeneous effects by applicant race and gender. Regression results provide a more streamlined manner to assess heterogeneity than, for instance, a large number of tables displaying results from McNemar’s tests.

Before proceeding to the results, we note that we favor LPMs over logit or probit models to facilitate interpretation, although we also replicated the analysis using a logit model with highly similar results (note: all of our models, including the logit models, can be re-estimated with our Stata do file and data archived on Open Science Framework). As Von Hippel (2015) explains, for probabilities in the range of 0.20 to 0.80, as is generally the case of the callback rates in our audit study, the LPM is a very close approximation to the logistic model, with both models fitting similarly well. In this case, one might favor the LPM, as we do here, because of its ease of interpretation (see also Pischke 2012).

For this analysis, our data is structured such that there is one record per applicant. The police vs. fire and police vs. code enforcement comparisons are pooled into the same data file, with police as the reference category. As two applicants applied for each position, one treatment and one control, we constructed a job ID indicator that is duplicated across the two applicants who applied for a given job. In our LPMs, we cluster our standard errors by these job IDs.

In the results displayed to follow, we pooled together observations from both sampled cities, Boston and Philadelphia. In supplemental analyses, we also estimated models separately for each city, with similar inferences (results not shown, but code for estimation is available in our archived Stata do file).

In Table A.1, we first present results (Model 1) for the combination of prior profession and time period (i.e., pre- or post- the death of George Floyd). We are also interested in whether any stigmatization of the police varies by characteristics of the applicant such as race and gender. Accordingly, in Model 2 we include three-way interactions between the treatment, time period, and race. Model 3 similarly displays three-way interactions between the treatment, time period, and gender. Model 4 includes four-way interactions across all factors (prior profession, time period, race, and gender). With the exception of one marginally significant coefficient in Model 4, for the time period x race interaction, we do not find evidence of statistically significant interaction effects.

For interpretation, the intercept and various combinations of coefficients can be summed in order to compute the predicted probability of a job call-back for a specific group. In our archived Stata do file, we include the *margins* command for this purpose. To illustrate, the predicted probability of receiving an affirmative call-back from a prospective employer in the pre-Floyd period is 0.193 for a Black male police officer (0.200 intercept and -0.007 gender coefficient) and 0.268 for a White male officer (0.200 intercept, -0.007 gender coefficient, 0.024 race coefficient, and 0.051 race x gender coefficient).

Given the marginally significant time period by race interaction in Model 4 as well as research evidence that White police officers are much more likely to use force against suspects, particularly in predominantly Black neighborhoods (e.g., Hoekstra and Sloan 2020), in Figures A.1 and A.2 we compare the gap in employer response rates to White relative to Black applicants across time period (A.1 for male applicants and A.2 for female). For instance, the left most columns in Figure A.1 reveal that in the time period before the killing of George Floyd, White police officers were estimated to receive an affirmative response to a job application 7.5 percentage points more often than Black police officers (a 0.268 callback rate for Whites vs. 0.193 for Blacks). Following the killing of Mr. Floyd, the gap declines to 3.2 percentage points (0.297 vs 0.265).

In all combinations presented in Figures A.1 and A.2, we see that the White advantage in employer responses narrows, even to the point of producing a Black advantage in employer response for all female applicants and for male code enforcement applicants. Thus, whereas we do not find evidence of a penalty in employer response against former police officers in the period of social unrest from police violence (i.e., see results in Tables 2 and 3 as well as Model 1 of Table A.1), we do observe some preliminary evidence that, for all professions, White job applicants may be affected in the job market during a period of widespread attention to police violence initiated by a White officer (i.e., Derek Chauvin) against a Black victim (i.e., George Floyd). Future research should continue to examine the consequences of police violence, and the ensuing public attention, for the job market prospects of applicants from different races. This future research should give attention not only to the potential effects for former police officers in the job market, but job applicants in general.

**Additional References**

Hoekstra, M. & Sloan, C.W. (2020). Does race matter for police use of force? Evidence from 911 calls. NBER Working Paper 26774. Retrieved 4 January 2021, from: <https://www.nber.org/papers/w26774>.

Pischke, J.-S. (2012). Probit better than LPM? Retrieved December 30, 2020, from: <http://www.mostlyharmlesseconometrics.com/2012/07/probit-better-than-lpm/>.

Von Hippel, P. (2015). Linear vs. Logistic Probability Models: Which is Better, and When? Retrieved December 30, 2020, from: <https://statisticalhorizons.com/linear-vs-logistic>.

**Figure A.1 Percentage Point Gap in Employer Response Probabilities between White Applicants and Black Applicants, by Profession and Time Period (Male Applicants Only)**

**Figure A.2 Percentage Point Gap in Employer Response Probabilities between White Applicants and Black Applicants, by Profession and Time Period (Female Applicants Only)**
